# Carbon replicas reveal double stranded structure of tight junctions in phase-contrast electron microscopy

**DOI:** 10.1038/s42003-019-0319-4

**Published:** 2019-03-12

**Authors:** Evan S. Krystofiak, J. Bernard Heymann, Bechara Kachar

**Affiliations:** 1 Laboratory of Cell Structure and Dynamics, National Institute on Deafness and Other Communication Disorders, National Institutes of Health, Bethesda, MD 20892 USA; 2 Laboratory of Structural Biology Research, National Institute of Arthritis, Musculoskeletal and Skin Diseases, National Institutes of Health, Bethesda, MD 20892 USA; 3 Present Address: Cell Imaging Shared Resource, Department of Cell and Developmental Biology, Vanderbilt University School of Medicine, Nashville, TN 37240 USA

## Abstract

Replica-based freeze-fracture and freeze-etching electron microscopy methods provide surface topography information, particularly suited to studying membrane protein complexes in their native context. The fidelity and resolution of metal replicas is limited by the inherent property of metal atoms to crystallize. To overcome the limitations of metal replicas, we combined amorphous carbon replicas with phase-contrast electron microscopy. Using this approach, tight junction intramembrane fibrils were shown to have a double stranded morphology.

## Abstract (web summary)

Krystofiak et al. combined carbon replicas with phase-contrast electron microscopy to overcome the limited fidelity and resolution of metal replicas. This method reveals a double stranded morphology of the tight junction intramembrane fibrils, demonstrating its superiority over conventional freeze-fracture methods.

## Introduction

Metal replica-based electron microscopy methods are widely used to examine surface topography of biological structures. Among these methods, freeze-fracture and freeze-etching occupy a unique niche providing surface topography views of membranes and other cellular structures in their native context, which is typically inaccessible by other techniques such as atomic force microscopy. However, the effectiveness of the metal replica to encode topographic features is limited by the inherent property of metal atoms to coalesce and form nanocrystals as they are deposited on the sample surface^1^. The production of replicas with finer texture using different metals or metal alloys has long been sought-after but with limited results^1–3^. Alternatively, Muller et al.^4^ showed that thin amorphous carbon replicas can encode higher resolution details of biological surfaces than those provided by metal replicas. However, the technique has not been widely adopted, mainly due to the poor electron scattering properties of carbon compared to heavy metals, thus limiting their practical use. Here, we demonstrate that carbon replicas, when combined with phase contrast electron microscopy procedures including the hole-free or Volta phase plate^5^, can provide practical improvements in the biologically relevant resolution of freeze-etching and freeze-fracture methods.

## Results

**Fig. 1:**
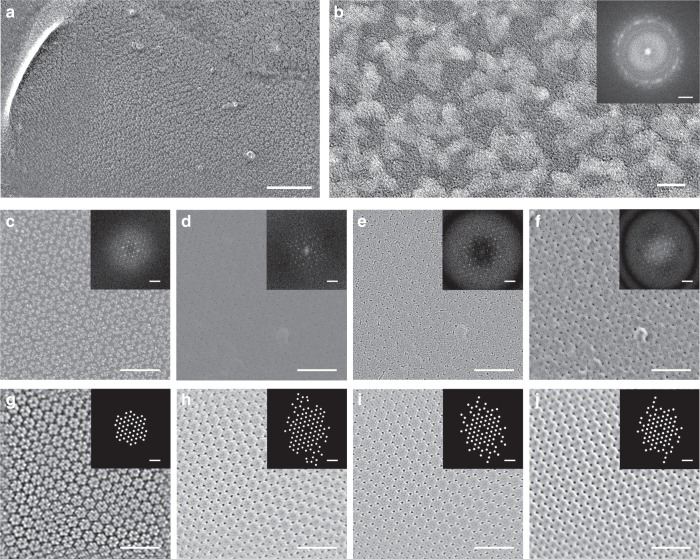
Platinum crystallization in conventional freeze-fracture replicas and resolution improvement of amorphous carbon replicas. **a** Electron micrograph of a conventional rotary shadowed freeze-etch of uroplakin complexes on the luminal surface of the bladder epithelium. **b** High magnification micrograph of a single uroplakin complex and its power spectrum (inset). **c**–**f** Direct comparison of uroplakin complex freeze-etch replicas using conventional rotary shadowing (**c**) and carbon shadowing imaged at near focus (**d**), defocus (**e**) or with a phase plate (**f**). Power spectra (insets) show the diffraction spots arising from uroplakin lattice. **g**–**j** The images in **c**–**f** were Fourier filtered to highlight the lattices in a conventional replica (**g**), and carbon replicas imaged near focus (**h**), defocus (**i**), or with a phase plate (**j**). The spot positions are highlighted in the spatial frequency mask (insets in **g**–**j**). These masks were used to generate the filtered images. Scale bars = 100 nm for **a**, 5 nm for **b**, 2 nm^−1^ for **b** inset, 50 nm for **c**–**j**, and 0.2 nm^−1^ for the power spectrum insets in **c**–**j**

We compared the biological resolution of platinum versus carbon replicas using the well-ordered, hexagonal-packed uroplakin complex on the luminal surface of the bladder epithelium^6–8^. Mouse bladders were freeze-etched at −100 °C and replicas of the surfaces were produced using low-angle (22°) rotary shadow using either platinum or carbon as the shadowing material. The hexagonally packed uroplakin complexes^6^ can be visualized at low magnification in platinum replicas of the luminal surface of the bladder (Fig. 1a). High magnification images of these replicas show polycrystalline aggregates decorating each uroplakin complex (Fig. 1b). Despite the irregular appearance of these polycrystals, the computed diffraction pattern shows the expected hexagonally packed uroplakin complexes^6^ (Fig. 1c). Carbon replicas also display the expected hexagonal packing of the complexes, but at very low amplitude contrast even when using a direct electron detection camera and frame averaging (Fig. 1d). The signal-to-noise ratio of the images was greatly improved by increasing contrast through defocusing (Fig. 1e). To generate contrast near focus we imaged the replicas using a hole-free phase plate^5,9^ (Fig. 1f).

We assessed the resolution of the platinum and carbon replicas by examining the highest spatial frequencies observed in the computed power spectrum (Fast Fourier Transforms) of flat regions of uroplakin complexes. Conventional platinum replicas produced spatial frequencies out to ~3.0 nm (Fig. 1c, inset), while the information in carbon replicas extended to ~1.9 nm in all three imaging conditions (Fig. 1d–f, insets). The anisotropy of the power spectra of the carbon replicas can be attributed to the uroplakin complexes not always being uniformly flat in relation to the replica surface, i.e. perpendicular to the carbon evaporation source or to the microscope electron beam. The repeating structural features of the uroplakin complexes can be averaged and better visualized by Fourier filtering (Fig. 1g–j). Fourier filtering of carbon replicas in near focus, defocus, and phase plate imaging conditions show the hexagonally packed uroplakin complexes as well as details within each complex (Fig. 1h–j). For example, the Fourier filtered carbon replica images showed the outer arms of the uroplakin complex as well as a distinct triangular-shaped gap between protein complexes that was not well-resolved in the platinum replicas. The lower resolution of platinum replicas appears to be mainly caused by platinum crystallization (see the clusters in Fig. 1b). These platinum nanocrystals are presumably a consequence of island type growth as the platinum preferentially interacts with itself rather than the frozen biological surface immediately following deposition. Additionally, platinum nanocrystals can have preferential nucleation sites on the sample surface, creating what has been referred to as platinum “decoration”^1^. It is likely that the preferential decoration of the central portion of the uroplakin complex (Fig. 1c) obscures the outer arms of the complex. The combination of uniform deposition and the non-crystalline nature of the carbon replicas produce a higher fidelity “cast” of the biological surface when compared to conventional platinum replicas.

To further evaluate the ability of carbon replicas to resolve structural details of a well-characterized intramembrane molecular complex, we examined connexin hemichannels. We prepared carbon replicas of freeze-fractured HEK293T cells expressing connexin 26 and imaged the freeze-fractured intramembrane hemichannels near focus, defocused, and with a phase plate (Supplementary Fig. 1a–c). We employed two-dimensional class averaging of the individual connexin 26 hemichannels using Bsoft^10^ to computationally reduce the noise and examine the protein complex topography. The average image depicts a hexameric topography with a carbon shadow around a central hole (Supplementary Fig. 1d, e). The connexin 26 hemichannel structure (PDBID: 5ERA)^11^ fits within this carbon cast (Supplementary Fig. 1f, g). This suggests that freeze-fracture carbon replicas and particle averaging could be used to more accurately identify the symmetry, surface topography, and dimensions of protein complexes.

We used our carbon replica method to examine the molecular architecture of tight junction intramembrane fibrils. Tight junctions are specializations of the plasma membrane at cell–cell contacts consisting of a network of intramembrane fibrils. These fibrils have been observed by freeze-fracture for decades^12–14^, but details of the structural and molecular architecture of the fibrils have not been resolved. The backbone of each intramembrane fibril is made of a linear polymer of claudins^15^. Various models of this linear claudin polymer have been proposed including an anti-parallel double-strand of claudins in each fibril^16^ as well as single-strand models with a diversity of different claudin–claudin interfaces^17^. While it has been argued that the width of the tight junction intramembrane fibril seen in metal-based freeze-fracture replicas approximates the dimensions of the double-strand model^16^, there is no direct structural evidence for the double-strand model.

**Fig. 2:**
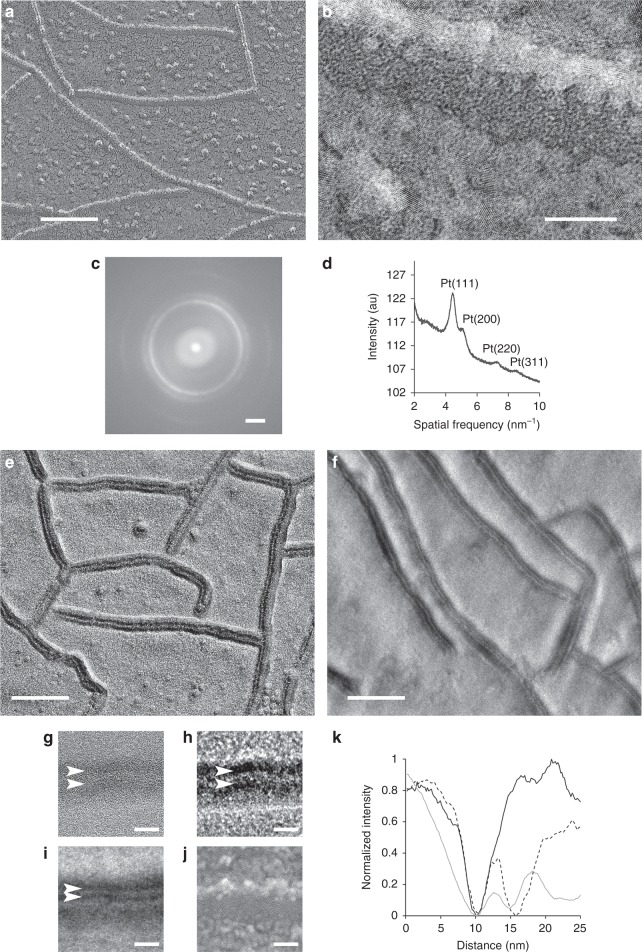
The double-strand structure of tight junction fibrils and distinct structural organization. **a** Claudin-11 fibrils visualized by unidirectional platinum shadowing freeze-fracture of transfected HEK293T cells. **b** Higher magnification of a single claudin fibril demonstrating platinum decoration. **c** The power spectrum and calculated radial intensity profile (**d**) show characteristic polycrystalline rings consistent with the spatial frequencies associated with crystalline platinum. Unidirectional carbon freeze-fracture of HEK293T cells expressing claudin-11 and connexin 26 imaged using phase plate (**e**) or single sideband imaging (**f**). Carbon replicas feature a double-lined morphology (arrowheads) that can be observed at near focus (**g**), with phase plate (**h**), and with single sideband imaging (**i**), while platinum replicas show a single-lined morphology (**j**). **k** Average intensity profiles through claudin-11 strands in platinum replicas (solid black) and carbon replicas imaged using a phase plate (dotted) and single sideband (solid gray). Scale bars = 100 nm for **a, e**, **f**, 10 nm for **b**, **g**–**j**, 2 nm^−1^ for **c**

In conventional, unidirectional-shadowed, platinum replicas of claudin-11-expressing HEK293T cells, the tight junction fibrils appeared as a single electron dense line with a single shadow in the direction of the platinum deposition (Fig. 2a). Rotary shadowed platinum replicas (Supplemental Fig. 2) also show fibrils with a single-strand appearance. Interestingly, the deposition of platinum on each side of the fibril better outlines the fibril width. The measured width of the fibril in the rotary shadowed platinum replicas was 6.9 ± 0.8 nm (*n* = 100 width measurements on three separate fibrils), which also approximates the dimensions of the double-strand model^16^. Close-up views of all platinum replicas showed nanocrystals (Fig. 2b). The power spectra of these crystals is consistent with nanocrystalline platinum reflections observed by selected area electron diffraction^18^ and with the observed atomic lattice spacings matching the known platinum lattice constant^19^.

To verify if the improved resolution of the carbon replicas could provide direct visualization of the double-strand structure of the tight junction fibril, we imaged unidirectionally shadowed, carbon replicas of tight junction fibrils using either a phase plate (Fig. 2e) or an adaptation of single sideband (Fig. 2f) phase contrast^20,21^. Both imaging methods (detailed in the Methods section) showed tight junction fibrils as two distinct parallel lines each alternating between light and dark contrast, consistent with a double-strand structure (Fig. 2g–i). Supplementary Movie 1 illustrates the increase in phase contrast and signal to noise highlighting the double-strand structure of the fibril as the phase plate is conditioned. Intensity profiles through claudin strands were created by averaging straightened strands using Bsoft^10^ (Fig. 2k). The average distance between the intensity minima of the two strands across the fibril was 5.3 nm on strands imaged with a phase plate and 4.6 nm by single sideband imaging. This supports the Suzuki double-strand model, predicted to have an overall diameter of 6 nm^16^ when taking into account the plastic deformation of fibrils during the fracture process^13^.

Tight junction fibrils can bend, branch, or anneal to form complex two-dimensional networks^22,23^. It is difficult to determine the nature of the interactions between fibrils in platinum shadowed replicas. In our carbon replicas, the double-strand structure is maintained at high curvatures and sharp bends (Supplementary Fig. 3a), consistent with an intrinsic flexibility of tight junction fibrils^23^. In some cases, clusters of protein particles are apparent near, at the point of contact between strands, or even appearing to integrate into the claudin fibril itself (Supplementary Fig. 2a–c). These clusters may play a role in the formation or stabilization of the points of contact or annealing of fibrils to form the characteristic two-dimensional tight junction networks. At some tight junction fibril contacts or intersections, at least one of the strands appears to be continuous across the intersection, suggesting that the double-stranded fibrils can form branches (Supplementary Fig. 3b).

## Discussion

Amorphous carbon is capable of encoding more biologically relevant details than platinum shadowed replicas, at the expense of amplitude contrast. The use of contrast-enhancing techniques largely overcome this limitation and allows observation of protein complexes in cellular membranes with minimal disruption of their native configuration. This is best done using a phase plate. In lieu of a phase plate, applying defocus and the type of single sideband imaging employed in these experiments can be performed on nearly any microscope. Carbon replicas are useful for imaging membrane protein complexes in their native membrane context, particularly if the complexes cannot be purified or otherwise prepared for cryoEM-based methods. We show that such improved topographic information can reveal new structural features of membrane-embedded protein complexes.

Using carbon replicas we provide direct evidence for a double-stranded structure of the tight junction intramembrane fibrils in their native location. Direct visualization of this fundamental structural feature of the tight junction fibril can help elucidate how the fibrils are formed and remodel, what leads to their intrinsic flexibility, and the nature of the inter-fibril contacts, branching, and annealing mechanisms.

Our particle averaging of connexin 26 hemichannels demonstrates that carbon replicas are amenable for aligned averaging and these averages can provide surface details of protein oligomers. We explored the use of carbon replicas for freeze-fracture and freeze-etching; however, there are other techniques that could potentially benefit from the improved resolution. Of particular interest are cell unroofing^24,25^, used to examine the proteins associated with the cytoplasmic side of the plasma membrane, membrane extracted cells^26^, and rotary molecular shadowing^27^.

## Methods

### Constructs and cell preparation

HEK293T cells were grown under standard conditions in Dulbecco's modification of Eagle medium supplemented with 10% fetal bovine serum. Cell cultures, at 70% confluency, were transfected with GFP-claudin-11 (pEGFP C2) and mCherry-connexin 26 constructs using Lipofectamine LTX (Invitrogen) according to the manufacturer’s instructions. mApple-Cx26-7 was a gift from Michael Davidson (Florida State University). After incubation with transfection reagent for 6 h the cells were washed extensively and allowed to grow to confluency. Samples were fixed in 2% glutaraldehyde and slowly equilibrated with 30% glycerol for cryoprotection. The cells were rapidly frozen using a LifeCell “slam freezer” by contact with a sapphire surface precooled to −184 °C. The frozen samples were stored in liquid nitrogen until freeze-fracture.

### Tissue preparation

The care and use of animals conformed to NIH guidelines and was approved by the Animal Care and Use Committee at the National Institute on Deafness and Other Communication Disorders (protocol #1215). Bladder tissue was prepared as previously described^6^. In brief, adult C57BL/6 mice were euthanized by CO_2_ asphyxiation and then decapitated. Bladders were rapidly isolated, inflated, and immersed in 2.5% glutaraldehyde in HBS for 2 h. The bladders were extensively rinsed in double-distilled water over a 24-h period. The samples were cut into small pieces and mounted on gelatin cushions, and then frozen as above.

### Freeze-fracture

Samples were freeze-fractured in a Balzers 301 freeze-fracture unit (Balzers Union) equipped with Cressington EB602PC electron-beam system (Cressington), turbo-drag pump (Pfieffer), and scroll pump (Edwards). For platinum replicas, the shadowing gun was loaded with a 2 mm predrilled carbon rod loaded with a 1.6 mm platinum pellet. The tip of the platinum pellet was positioned at the center of the coiled tungsten filament. For carbon replicas the shadowing electron-beam source was loaded with a 2 mm carbon rod with the rod tip positioned at the center of the coiled tungsten filament. Rotary shadowing of connexin 26 and claudin-11 samples was performed at 20° while unidirectional shadowing of tight junction samples was performed at 45° relative to the sample. All replicas were stabilized with a secondary carbon coating, using the overhead electron-beam gun loaded with a 3 mm carbon rod with the tip also positioned at the center of the coiled tungsten filament.

Samples were loaded into the freeze-fracture machine cooled to −180 °C. After a vacuum of better than 5 × 10^−6^ mbar was reached the knife was cooled and once the vacuum reach the 10^−7^ mbar range the temperature raised to −110 °C. Samples were freeze-fractured by the standard Balzers machine’s microtome and immediately shadowed. For conventional metal replicas, freeze-fractured or freeze-etched samples were shadowed with platinum using 75 mA at 2 kV for 5 s (2–3 nm nominal thickness) and followed by stabilization with carbon using 90 mA at 2 kV for 6 s, deposited perpendicular to the sample (6–8 nm nominal thickness). Carbon replicas were produced in a similar manner as platinum replicas but sequentially shadowed and overhead stabilized with a pure carbon 90 mA at 2 kV for 6 s per electron beam gun for a nominal thickness of 6–10 nm.

All replicas were floated from the biological surfaces by carefully placing the samples at a low angle into a 6% sodium hypochlorite solution (Clorox). The samples were kept floating on the surface of the sodium hypochlorite for at least 1 h, then washed in double-distilled water. The replicas were collected by placing 300 mesh hexagonal copper grids (Electron Microscopy Sciences) dull side down onto the floating replica pieces and dried by blotting on filter paper, taking care not to directly touch the replica.

### Freeze-etching

Bladder samples were freeze-fractured as described above and then allowed to etch at −100 °C for 10–15 min under the microtome. After etching, the surface was coated with carbon by an initial 20° rotatory shadowing followed by 90° carbon evaporation as described above.

### Electron microscopy imaging

Samples were imaged at 200 kV on a JEOL 2200FS microscope (JEOL, Peabody, MA) equipped with a field emission gun, an in-column energy filter, and K2 direct electron detector (Gatan, Pleasanton, CA). All micrographs were acquired with zero-loss energy filtering, using the counting mode of the K2 camera and computed drift correction by Digital Micrograph (Gatan, Pleasanton, CA).

### Phase plate and single sideband imaging

The JEOL 2200FS microscope (JEOL, Peabody, MA) was equipped with a hole-free phase plate in the back focal plane of the objective lens. To produce contrast the C2 lens current was adjusted to place the beam crossover on the phase plate (in the back focal plane) and with parallel sample illumination. The irradiated area of the phase plate film was allowed to develop a Volta potential^5^ by constant exposure to the electron beam until the contrast of the sample stabilized (Supplementary Movie 1). Phase plate micrographs were imaged with minimal defocus.

Single sideband imaging involved positioning the edge of the objective aperture in the primary beam. The C2 lens was adjusted so that crossover occurred at the back focal plane of the microscope in the same manner as the phase plate.

### Image processing

Frame alignment, fast Fourier transforms, and Fourier filtering were performed using Digital Micrograph (Gatan, Pleasanton, CA). Power spectra were generated by the fast Fourier transform function in Digital Micrograph. Fourier filtering masks were created to pass high-intensity spatial frequencies. Fourier spot masks were placed over spatial frequencies that followed the hexagonal packing of the uroplakin complex and had at least 30% higher intensity than the local mean. An inverse FFT was applied to the masked FFTs to create the Fourier filtered real space images. Images were analyzed in FIJI^28^. Radial profile of the power spectra were generated using the Radial Profile plugin available on the ImageJ plugin repository (https://imagej.nih.gov/ij/plugins/). Brightness and contrast were adjusted in Photoshop (Adobe, San Jose, CA).

### Single particle averaging

The Bsoft package was used for all steps in the replica single particle averaging^10,29^. Connexin particles were manually boxed, normalized, and background subtracted. Only particles with top views were considered (*n* = 115). An isotropic ring roughly the size of the particles was used as an initial reference. The particles were iteratively aligned using the C6 symmetrized average of the previous alignment until the average converged to a consistent result after seven iterations. The connexin 26 crystal structure (PDBID: 5ERA)^11^ was depicted using UCSF Chimera^30^; connexin 26 residues below the P-face were truncated to match the freeze-fracture structure.

### Claudin strand profiles

The filament processing capability of the Bsoft package was used to calculate claudin strand profiles^10^. Claudin-11 fibrils (~200 nm in length) were traced as filaments in Bshow and the average profiles recorded. The strand straightening is done by fitting a Catmull-Rom spline through the filament nodes^31^.

### Reporting Summary

Further information on experimental design is available in the Nature Research Reporting Summary linked to this article.

## Supplementary information

Description of Additional Supplementary Files

Supplementary Information

Supplementary Movie 1

Reporting Summary

## Data Availability

All data in support of the findings of this study are available from the corresponding author by reasonable request.
