# Supplementary material for: Carbon replicas reveal double stranded structure of tight junctions in phase-contrast electron microscopy
Source: Commun Biol. 2019 Mar 12;2:98. doi: 10.1038/s42003-019-0319-4 (PMC6414538; doi:10.1038/s42003-019-0319-4)
Supplement: Supplementary file 1 — Description of Additional Supplementary Files [file 42003_2019_319_MOESM1_ESM.docx]

Supplementary Movie 1: Establishment of phase-contrast during conditioning of the hole-free phase plate. Time series showing images of a freeze-fracture carbon replica of HEK293T cells transfected with claudin 11 as the phase plate is conditioned by the electron beam. The topography of the claudin 11 strands and membrane proteins becomes more apparent as the contrast increases with time. Scale bar = 100 nm.
