# Supplementary material for: Carbon replicas reveal double stranded structure of tight junctions in phase-contrast electron microscopy
Source: Commun Biol. 2019 Mar 12;2:98. doi: 10.1038/s42003-019-0319-4 (PMC6414538; doi:10.1038/s42003-019-0319-4)
Supplement: Supplementary file 2 — Supplementary Information [file 42003_2019_319_MOESM2_ESM.pdf]

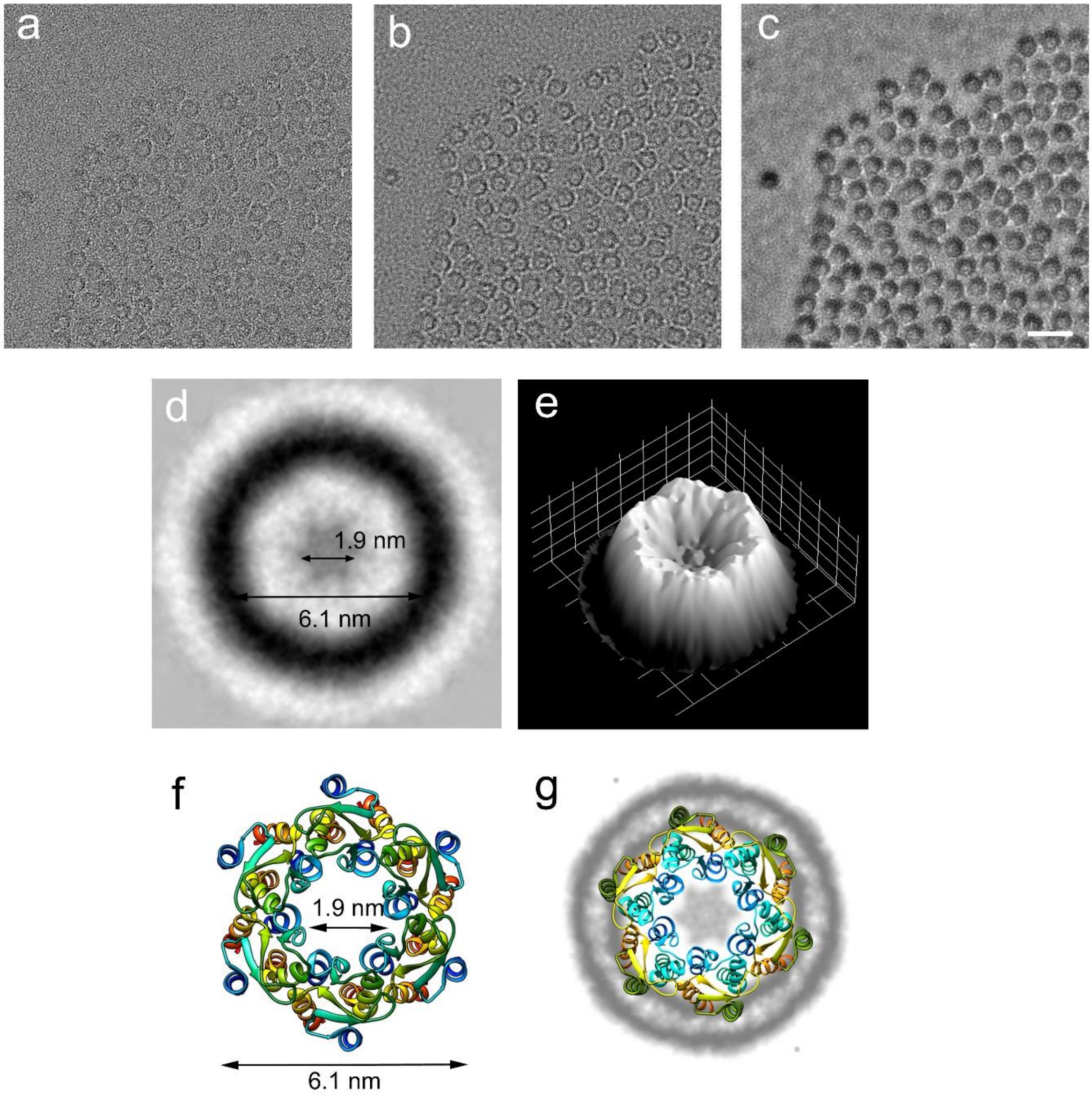

Supplementary Figure 1: Connexin 26 freeze-fracture and averaging. **a-c** Electron micrographs showing a freeze-fracture carbon replica of a connexin 26 hemichannel plaque taken at near focus **a**, -2 µm defocus **b**, and near focus with a phase plate **c**. **d** The two-dimensional, six-fold symmetrically averaged connexin 26 hemichannel projection, (n = 115 particles). **e** Three-dimensional surface plot of the projected average. **f** The crystal structure of the connexin 26 hemichannel showing the approximate diameter of the pore and edge of the hemichannel (PDBID: 5ERA)[11]. **g** The connexin 26 hemichannel superimposed over the two-dimensional average. Scale bar = 20 nm for **a-c**, 2 nm for **d**,**e**,**g**.

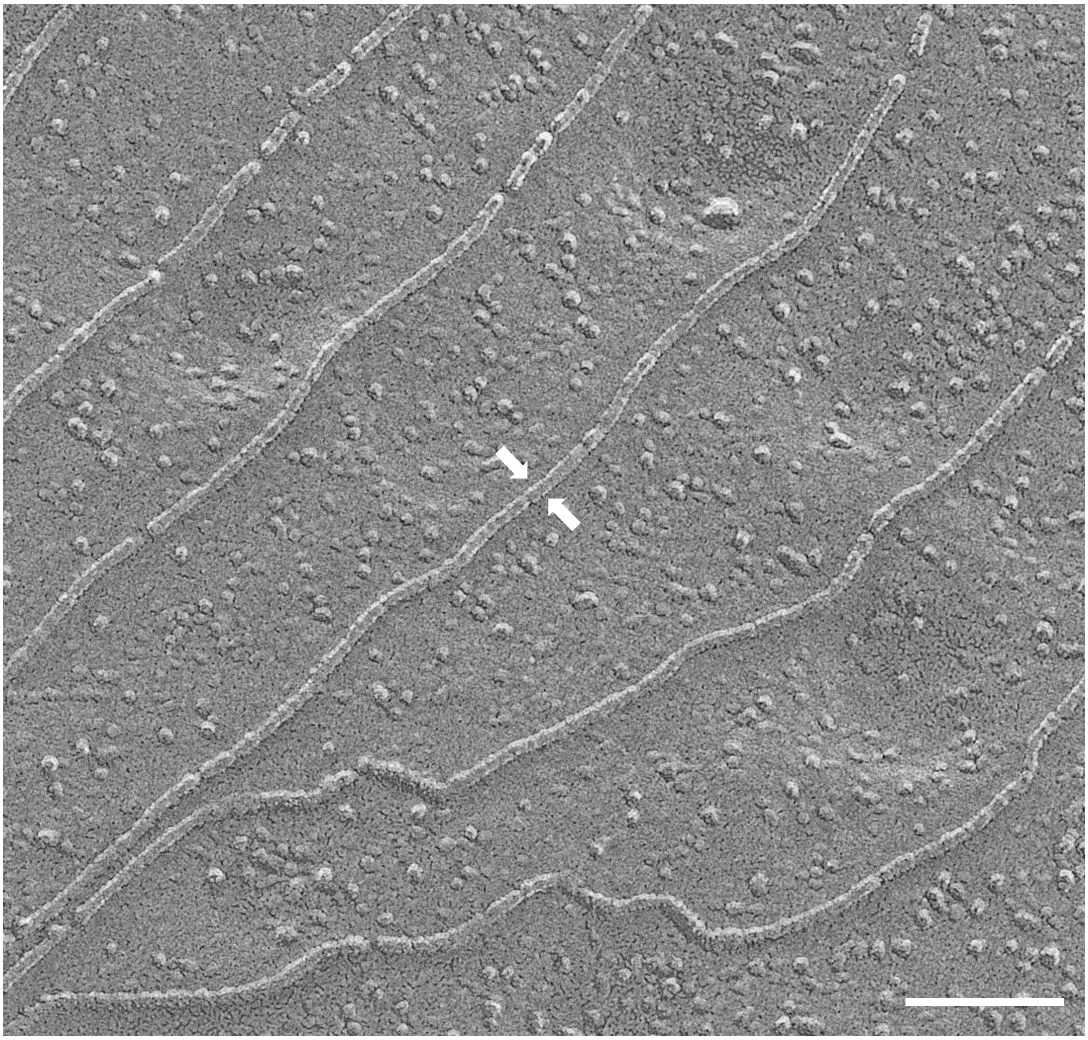

Supplementary Figure 2: Platinum rotary shadowing freeze-fracture replicas preserve the claudin fibril width. Claudin 11 fibrils visualized by platinum rotary shadowing freeze-fracture of transfected HEK293T cells. The platinum shadow highlights both sides of the strands (arrows) but does not reveal additional structural details. Scale bar = 100 nm.

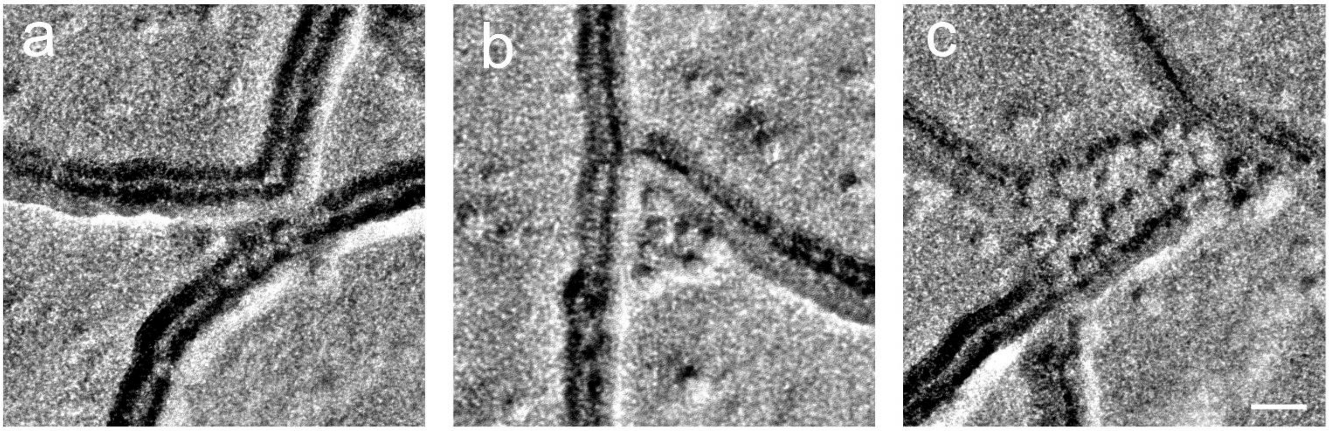

Supplementary Figure 3: Claudin strand morphologies. **a-c** Electron micrographs of carbon freeze-fracture replicas of HEK293T transfected with claudin 11 and connexin 26 imaged with a phase plate, showing a sharp bend or kink in the claudin strand **a**, a claudin-11 branch point with a cluster of protein particles **b**, and a larger cluster of protein particles inside a claudin-11 strand network **c**. Arrowheads indicate protein particles associated with claudin 11 strands at points of contact or annealing. Scale bar = 20 nm.
